# Nanoparticulate matter exposure results in neuroinflammatory changes in the corpus callosum

**DOI:** 10.1371/journal.pone.0206934

**Published:** 2018-11-05

**Authors:** Robin Babadjouni, Arati Patel, Qinghai Liu, Kristina Shkirkova, Krista Lamorie-Foote, Michelle Connor, Drew M. Hodis, Hank Cheng, Constantinos Sioutas, Todd E. Morgan, Caleb E. Finch, William J. Mack

**Affiliations:** 1 Zilkha Neurogenetic Institute, Keck School of Medicine, University of Southern California, Los Angeles, California, United States of America; 2 Department of Neurosurgery, Keck School of Medicine, University of Southern California, Los Angeles, California, United States of America; 3 Department of Civil and Environmental Engineering, Viterbi School of Engineering, University of Southern California, Los Angeles, California, United States of America; 4 Leonard Davis School of Gerontology, University of Southern California, Los Angeles, California, United States of America; Telethon Institute for Child Health Research, AUSTRALIA

## Abstract

Epidemiological studies have established an association between air pollution particulate matter exposure (PM2.5) and neurocognitive decline. Experimental data suggest that microglia play an essential role in air pollution PM-induced neuroinflammation and oxidative stress. This study examined the effect of nano-sized particulate matter (nPM) on complement C5 deposition and microglial activation in the corpus callosum of mice (C57BL/6J males). nPM was collected in an urban Los Angeles region impacted by traffic emissions. Mice were exposed to 10 weeks of re-aerosolized nPM or filtered air for a cumulative 150 hours. nPM-exposed mice exhibited reactive microglia and 2-fold increased local deposition of complement C5/ C5α proteins and complement component C5a receptor 1 (CD88) in the corpus callosum. However, serum C5 levels did not differ between nPM and filtered air cohorts. These findings demonstrate white matter C5 deposition and microglial activation secondary to nPM exposure. The C5 upregulation appears to be localized to the brain.

## Introduction

Exposure to air pollution particulate matter (PM) is a potent generator of neuroinflammation in the central nervous system (CNS) [1, 2] and has been associated with decreased white matter volume and reduced cognition in older adults [3–5]. Murine studies suggest that particulate matter exposure results in myelin loss in the CA1 stratum oriens of young mice, consistent with myelin reduction classically evident with aging [6]. While multiple CNS cell types are implicated in the inflammatory response, microglia have critical roles in particulate matter-induced CNS injury [7]. Under physiologic conditions, microglial activation enables homeostatic phagocytosis and facilitates synaptic remodeling and brain maturation. These phagocytic mechanisms, however, are aberrantly triggered in a host of disease processes [8]. Studies have demonstrated that macrophages and microglia contribute to white matter injury in the setting of multiple sclerosis[9], periventricular leukomalacia, and amyotrophic lateral sclerosis[10]. Microglia propagate neuroinflammation through expression of pro-inflammatory cytokines and generation of reactive oxygen species[11]. When activated, microglia produce complement proteins[12, 13] and express complement-specific receptors, particularly C5aR (CD88) [12, 14–16]. In vitro studies of activated microglia demonstrate adherence and cytotoxicity to oligodendrocytes in the presence of complement factors[17]. The complement cascade, and principally the C5 anaphylatoxin, may play an important role in the pathogenesis of white matter inflammation following nanoparticulate matter (nPM) exposure.

This investigation examines the association between nPM exposure and white matter (corpus callosum) C5 deposition in a murine model. Immunohistochemical analysis and ELISA studies explore the relationship between complement upregulation and the presence of reactive microglia.

## Materials and methods

### Protocol

All procedures utilized in this study were approved by the Institutional Animal Care and Use Committee (IACUC; protocol # 20235) of the University of Southern California and carried out in accordance with the Guide for the Care and Use of Laboratory Animals (NIH). All mice were male C57BL/6J mice (15–16 weeks of age; 24-29g) and housed in a barrier facility with free access to food and water on a 12-hour light dark cycle, except during the nPM/ filtered air exposures. The mice did not have access to food and water during the daily five-hour exposure periods.

#### Particulate matter collection

Collection of nPM (particles smaller than 0.2 μm in diameter) was conducted in an urban area in central Los Angeles, impacted mostly by traffic emissions[18, 19]. Briefly, urban nPM (aerodynamic diameter <200 nm) is collected at 400 L/min flow using a high-volume ultrafine particle sampler[19]. The sampler incorporates an ultrafine particle multiple rectangular (slit) geometry jet conventional impactor that removes particles larger than 0.2 μm, and the remaining nPM is collected on pretreated Teflon filters (8x10”, PTFE, 2 μm pore) and transferred into an aqueous suspension by 30 min soaking of filters in Milli-Q deionized water (resistivity, 18.2 MW; total organic compounds < 10 ppb; particle free;endotoxin levels < 1 units/mL; endotoxin-free glass vials), followed by vortexing (5 min) and sonication (30 min) for resuspension. No endotoxin is detected in these suspensions (*Limulus*amebocyteassay: LPS <0.02EU/ml). As a control, fresh sterile filters were sham extracted and stored. Aqueous nPM suspensions were pooled and frozen as a stock at –20°C, following recommended procedures by the US EPA, which show retention of chemical stability for ≥ 3 mo[20].

For mouse exposure, the nPM were re-aerosolized by an atomizer using compressed particle-free filtered air as discussed in detail in previous publications [1, 19]. During mouse exposure, the particle size and concentration were continuously monitored by a scanning mobility particle sizer (SMPS model 3080; TSI Inc., Shoreview, MN) in parallel with the animal exposure chambers. We maintained the average nPM mass concentration at approximately 330 (+/- 25) ug/m^3^- roughly twice that of busy roadways [21]. From the total of 15 l/min of aerosol flow generated, the majority (10 l/min) was drawn through the exposure chamber. The remaining 5 l/min was diverted to filters for particle collection and characterization. Teflon and quartz filters, sample concurrently the aerosol during exposure. The mass concentration of the nPM was determined by pre- and post- weighing the Teflon filter under controlled temperature and relative humidity. Inorganic ions [ammonium (NH_4_^+^), nitrate (NO_3_^–^), sulfate (SO_4_^2–^)] were analyzed by ion chromatography, and PM-bound metals/ trace elements were assayed by magnetic-sector inductively coupled plasma mass spectroscopy. Water-soluble organic carbon collected on the quartz filter was assayed by a GE-Sievers liquid analyzer (GE-Sievers, Boulder, CO). More details about the inorganic and organic compound contents of these samples have been previously described[1]. Exposures were conducted in temperature and air controlled sealed whole-body exposure chambers with adequate ventilation to minimize buildup of animal-generated contaminants[1, 22, 23].

#### Particulate matter exposures

Mice were group-housed with four mice in each cage and randomized to re-aerosolized nPM or filtered air exposure cohorts. Exposures occurred for five hours/day, three-days/ week for a 10-week period. The mice were humanely euthanized 72 hours after the last exposure. While under anesthesia with ketamine (80 mg/kg IP) and xylazine (10 mg/kg IP), serum (up to .22ml) was collected via a direct cardiac puncture. The mice were then transcardially perfused with PBS and heparin (5 U/mL) saline followed by a fixative solution (4% paraformaldehyde and .2% picric acid in .1 mol/L phosphate buffer). The brains were harvested and stored in paraformaldehyde for 24 hours at 4°C. Tissue was then dehydrated in ethanol (70%) and sent to pathology for paraffin embedding.

#### Immunohistochemistry

Immunohistochemical analysis was performed on paraffin embedded brain sections as described below:

Slides were deparaffined, and hydrated using a series of different alcohol concentrations (ranging from 100% to 70%). Antigen was retrieved with Dako target retrieval solution, dipped in 3% H_2_O_2_ for 10 min, and then blocked with serum. Slides were incubated overnight with a rabbit anti- glial fibrillary acidic protein (GFAP) antibody (diluted 1:10 000; Dako, Denmark) or a rabbit anti-ionized calcium-binding adapter molecule 1(IBA1) antibody (1:200; Wako, Japan). Subsequently, sections were treated with the appropriate biotinylated secondary antibody Vectastain Elite ABC kit (Vector Laboratories, Burlingame, California, USA) and visualized with diaminobenzidine (DAB). A LAS AF microscope (Leica, Germany) was used to capture images of the immunostained slices. NIH Image J software was employed to quantify the optical density of DAB signal for analysis (rsbweb.nih.gov/ij/). The number of GFAP and IBA-1 positive cells in one high powered field (40x) were counted in the left medial and right medial corpus callosum and the two values subsequently averaged. IBA-1/GFAP cells that exhibited double immunopositivity with DAPI were only counted. For IBA-1 analysis, only heavily stained cells larger than 5 μm were counted. Separately, mean cell density of IBA-1/GFAP positive cells was counted in the left medial and right corpus callosum and the two values were subsequently averaged. To assess microglia morphology, images were evaluated using Image J software following a protocol previously described [24]. Briefly, the corpus callosum was selected and the “Adjust Threshold” function was applied as the intensity threshold. Total cell size was measured at this intensity threshold without a size filter. Cell body size was measured by lowering the threshold 5 points and applying a size filter of 100 pixels using the “Analyze Particles” function. To calculate the overall size of the dendritic processes, cell body size was subtracted from total cell size. From this, the cell body to dendritic process size ratio was calculated.

#### Immunofluorescence

Similar to the protocol for IBA-1 and GFAP staining, slides were deparaffined, and hydrated using a series of alcohol concentrations (ranging from 100% to 70%). Antigen retrieval was achieved with Dako target retrieval solution, dipped in 3% H_2_O_2_ for 10 min, and then blocked with 5% donkey serum. Slides were incubated overnight with anti-C5 (mouse 1:50 Hycult Biotech, Netherlands; clone BB5.1), anti-CD88 (rat 1:200 Hycult Biotech, Netherland; HM1076) or rabbit complement component C5α (125kDa) antibody (1:50 Santa Cruz, SC-21941). Subsequently, sections were washed, incubated with secondary antibodies (Alexa Fluor 568, Invitrogen, Carlsbad, CA) for one hour, and nuclei were stained with DAPI (Invitrogen) for 10 minutes. Slides were mounted with Dako fluorescent mounting media, coverslipped, and then visualized using Zeiss 510 confocal microscopy and BZ-9000 fluorescent microscopy (Keyence, NJ). NIH Image J software was used to quantify immunofluorescence. The images were converted to 8-bit and adjusted to threshold to count the positive cells. Mean density area for C5, C5α and CD88 was measured in one high powered field (C5: 400x, C5α: 400x, CD88:200x) in the left and right medial corpus callosum and the two values were averaged. Protocols followed the NIH Image J user guide.

Double immunofluorescence staining was carried out following a similar protocol to what was described above. Slides were incubated overnight with mouse anti-C5 (diluted 1:50, Hycult Biotech, Netherlands, HM1073) and one of the three primary antibodies: 1) rabbit anti-glial fibrillary acidic protein (GFAP) (diluted 1:10,000, Dako, Denmark), 2) rabbit anti-ionized calcium-binding adapter molecule 1 (IBA1) antibody (1:200; Wako, Japan), or 3) rabbit anti-choline acetyltransferase (ChAT) antibody (diluted 1:150, MilliporeSigma, Germany). ChAT is a marker for choline acetyltransferase and can be used to identify cholingeric neurons [25]. Sections were then washed and incubated for one hour with one of the three secondary antibody solutions based on the previous primary antibody solution: 1) Alexa Fluor 800 and Alexa Fluor 647, 2) Alexa Fluor 800 and Alexa Fluor 647, or 3) Alexa Fluor 568 and Alexa Fluor 647 (Invitrogen, Carlsbad, CA). Nuclei were stained with DAPI (Invitrogen), slides were mounted with mounting media, and subsequently imaged following the above protocol. Three images were taken with different channels to visualize the different stains. NIH Image J software was used to merge the images using the “Image Calculator” function.

#### ELISA analysis

Serum was obtained by direct cardiac puncture at the time of euthanasia from nPM and filtered air groups. ELISA was performed for C5 (ηg C5 protein/ mg total protein) and TNF-alpha (ηg TNF-alpha protein/ mg total protein) according to manufacturer’s instructions (C5; Kamiya Biomedical Company, KT– 11775, Seattle, WA) (TNF-alpha; R&D Systems P134505, Minneapolis, MN).

### Statistical analyses

The differences between nPM and filtered air cohorts were analyzed with two-tailed unpaired Student’s t-tests. Data in the text are presented as mean±standard deviation. P ≤ .05 is considered statistically significant.

## Results

The mass concentration during the 150 hours of the exposure was 330 (±25) μg/m^3^ whereas the number concentration was 1.6 (±0.3) *10^5^ particles/cm^3^. Total Organic Carbon (TOC) was the most predominant chemical species, accounting for 68 (± 9)% of the total mass. The mass fractions of other trace elements and metals (in units of ng/μg of PM mass) are listed in the table below (Table 1). The size distribution of the exposure aerosol is presented below (Table 2) and is typical of particulate matter in an urban area impacted by traffic emissions[1].

**Table 1:** Mass fractions of trace elements and metals (ng/μg of PM mass) during exposure.

Metal / Trace Element	Mass Fraction
Na	36.88 ± 0.46
Mg	10.34 ± 0.02
Al	8.85 ± 0.04
S	37.69 ± 0.27
K	6.67 ± 0.07
Ca	33.28 ± 0.35
Ti	0.35 ± 0.04
V	0.04 ± 0.00
Cr	0.16 ± 0.00
Mn	0.33 ± 0.00
Fe	8.65 ± 0.07
Ni	0.26 ± 0.01
Cu	0.58 ± 0.01
Zn	2.98 ± 0.02
Pb	0.11 ± 0.00

**Table 2:** Size distribution of the exposure aerosol.

	Mean	Std. Dev.
Median (nm)	55.2	0.6
Mean (nm)	68	0.7
Geo. Mean (nm)	55.9	0.5
Mode (nm)	53.3	3.9
Geo. Std. Dev.	1.8	
Total Concentration (particles/cm^3^)	3.9E+05	3.3E+03

### Reactive microglia (IBA-1) and astrocytes (GFAP)

**Fig 1:**
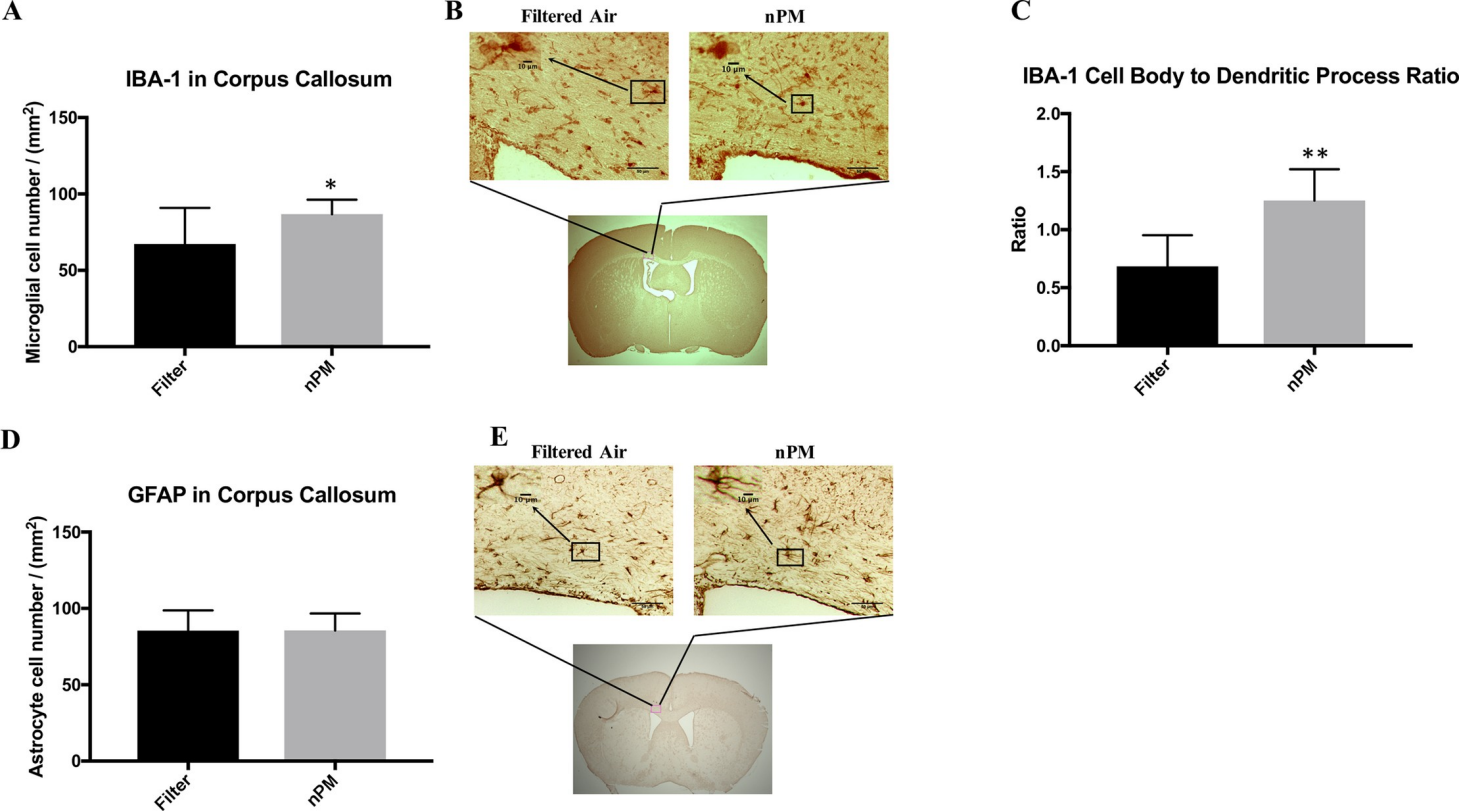
IBA-1 and GFAP staining for reactive glial cells in the medial corpus callosum of mice exposed to filtered air or nanoparticulate matter (nPM). (A) IBA-1 positive cell counts in each experimental group. The nPM group showed significantly greater microglial cell count (n = 8) compared to the filtered air group (n = 8, p = 0.047). * signifies p < 0.05. (B) Below; low magnification representation of region analyzed. Above; filtered air and nPM exposed mice stained for IBA-1 in the corpus callosum (40x). The upper left hand corner is a high magnification representation of a single cell. (C) IBA-1 cell body to dendritic process ratio in each experimental group. The nPM cohort had a significantly increased ratio (n = 8) compared to the filter group (n = 8, p < 0.001). ** signifies p < 0.001. (D) GFAP positive cell counts in each experimental group. There was no significant difference in astrocyte cell count between groups (p = 0.983) (E) Below; low magnification representation of region analyzed. Above; filtered air and nPM exposed mice stained for GFAP in the corpus callosum (40x). The upper left hand corner is a high magnification representation of a single cell. Error bars represent standard deviation. Scale bars are presented on the lower right corner of the images.

IBA-1 cell counts in the medial corpus callosum were increased 30% by exposure to nPM: filtered air (67.3 ± 23.6, n = 8) and nPM mice (86.9 ± 9.4, p = 0.047, n = 8) (Fig 1A and 1B). However, no differences existed in the medial corpus callosum for GFAP cell counts between filtered air (85.5 ± 13.2, n = 8) and nPM mice (85.6 ± 11.0, p = 0.98, n = 8) (Fig 1D and 1E). Further, IBA-1 cell density in the medial corpus callosum was increased 25% by exposure to nPM: filtered air (140.2 ± 7.9, n = 8) and nPM mice (177.9 ± 4.1, n = 8, p<0.001). No differences in cell density existed in the medial corpus callosum for GFAP cell density between filtered air (178.3 ± 5.9, n = 8) and nPM mice (179.3 ± 8.4, p = 0.78, n = 8). IBA-1 cell body to dendritic process size ratio was significantly increased following nPM exposure (1.25 ± 0.27, n = 8) compared to filtered air (0.68 ± 0.27, p < 0.001, n = 8) (Fig 1C).

### Complement deposition and receptor expression: C5, C5α, CD88

**Fig 2:**
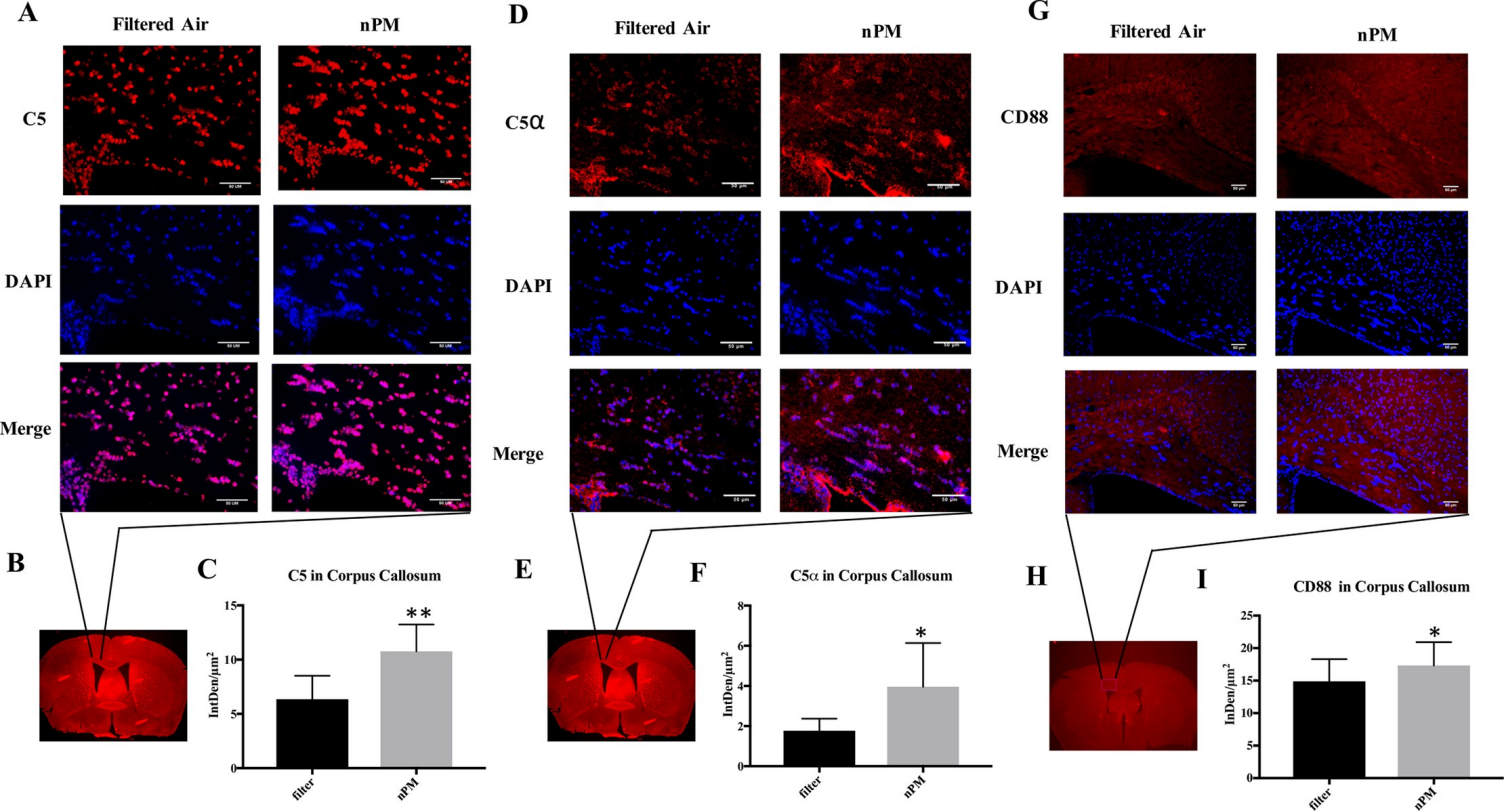
Immunohistochemical analysis of C5, C5α, and C5a receptor (CD88) in the corpus callosum of animals exposed to filtered air or nanoparticulate matter (nPM). (A) Filtered air (n = 8) or nPM (n = 8) exposed mice stained for C5 (red) in the corpus callosum. Nuclei (DAPI) are stained in blue (400x). (B) Low magnification representation of region analyzed. (C) C5 immunostaining in the corpus callosum was significantly higher in nPM exposed animals compared to the filtered air group (p = 0.001). (D) Filtered air (n = 8) and nPM (n = 8) exposed mice stained for C5α (red) in the corpus callosum. Nuclei (DAPI) are stained in blue (400x). (E) Low magnification representation of region analyzed. (F) C5α immunostaining in the corpus callosum of nPM exposed animals was significantly greater than in the filtered air group (p = 0.02). (G) Filtered air (n = 18) and nPM (n = 18) exposed mice stained for CD88 (red) in the corpus callosum. Nuclei (DAPI) are stained in blue (200x). (H) Low magnification representation of region analyzed. (I) CD88 immunostaining in the corpus callosum was significantly higher in nPM exposed animals compared to the filtered air group (p = 0.04). * signifies p< 0.05, ** signifies p ≤ 0.001. Error bars represent standard deviation. Scale bars indicate 50 μm.

Exposure to nPM caused 2-fold increases in C5 (Fig 2A–2C) and C5α (Fig 2D–2F) integrated density staining in the corpus callosum between mice in the filtered air (C5: 6.3 ± 2.2; C5α 1.8 ± 0.60, n = 8) and nPM groups (C5: 10.7 ± 2.5, p = 0.001; C5α: 3.96 ± 2.2, p = 0.02, n = 8). CD88 increased by 15% in the medial corpus callosum (Fig 2G–2I) between filtered air (14.9 ± 3.4, n = 18) and nPM mice (17.3 ± 3.6, p = 0.04, n = 18).

**Fig 3:**
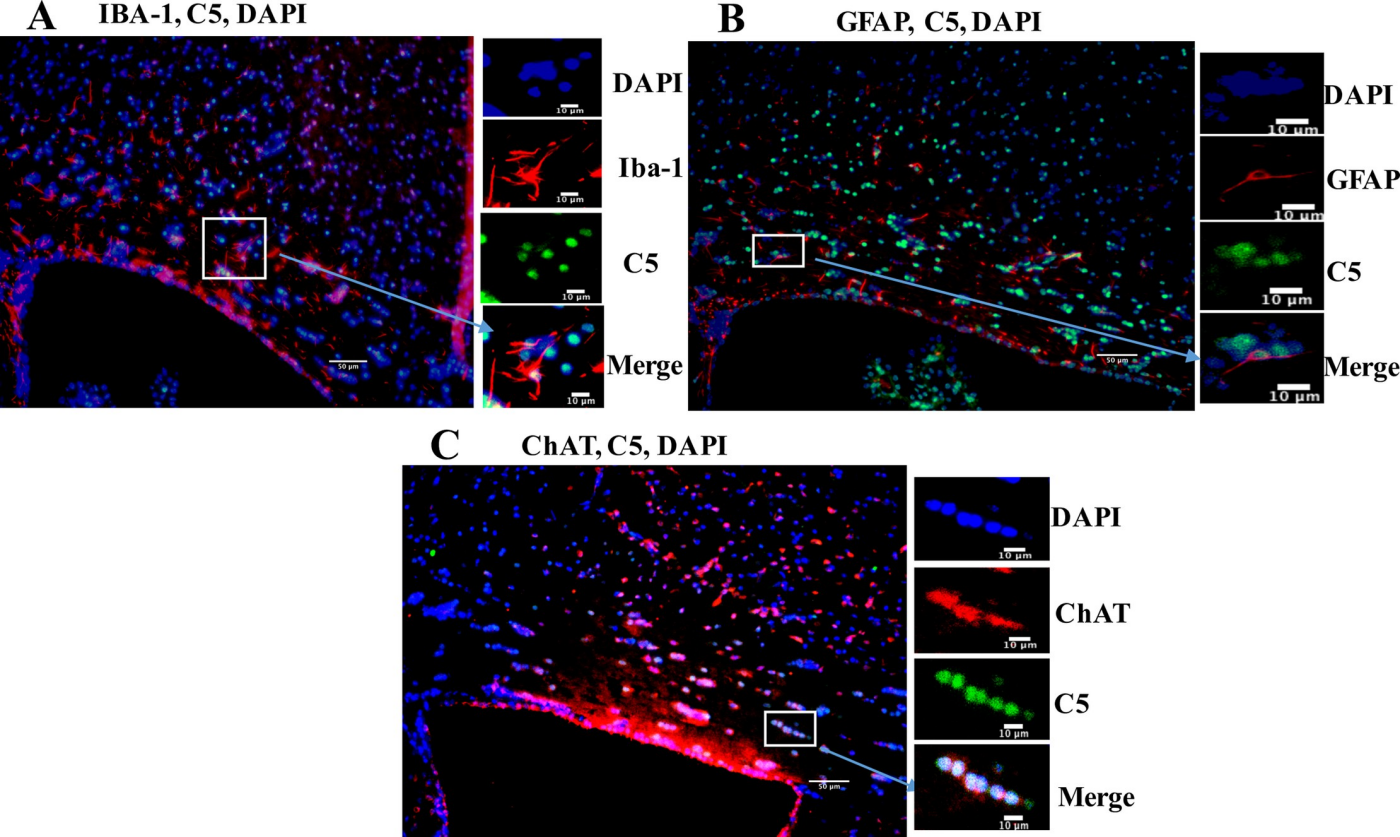
Double immunofluorescence staining of C5 with IBA-1, GFAP, or ChAT in the corpus callosum of animals exposed to nanoparticulate matter (nPM). (A) Co-staining of IBA-1 (red) with C5 (green) in the corpus callosum of a mouse exposed to nPM. Nuclei (DAPI) are stained in blue (200x). (B) Co-staining of GFAP (red) with C5 (green) in the corpus callosum of a mouse exposed to nPM. Nuclei (DAPI) are stained in blue (200x). (C) Co-staining of ChAT (red) with C5 (green) in the corpus callosum of a mouse exposed to nPM. Nuclei (DAPI) are stained in blue (200x). Scale bars are presented on the lower right corner of the images.

There was co-localization of C5 and ChAT (Fig 3C). There was a scattering of C5 on and nearby IBA-1 (Fig 3A) and GFAP positive cells (Fig 3B).

### Serum TNF-alpha levels and Serum C5 levels

**Fig 4:**
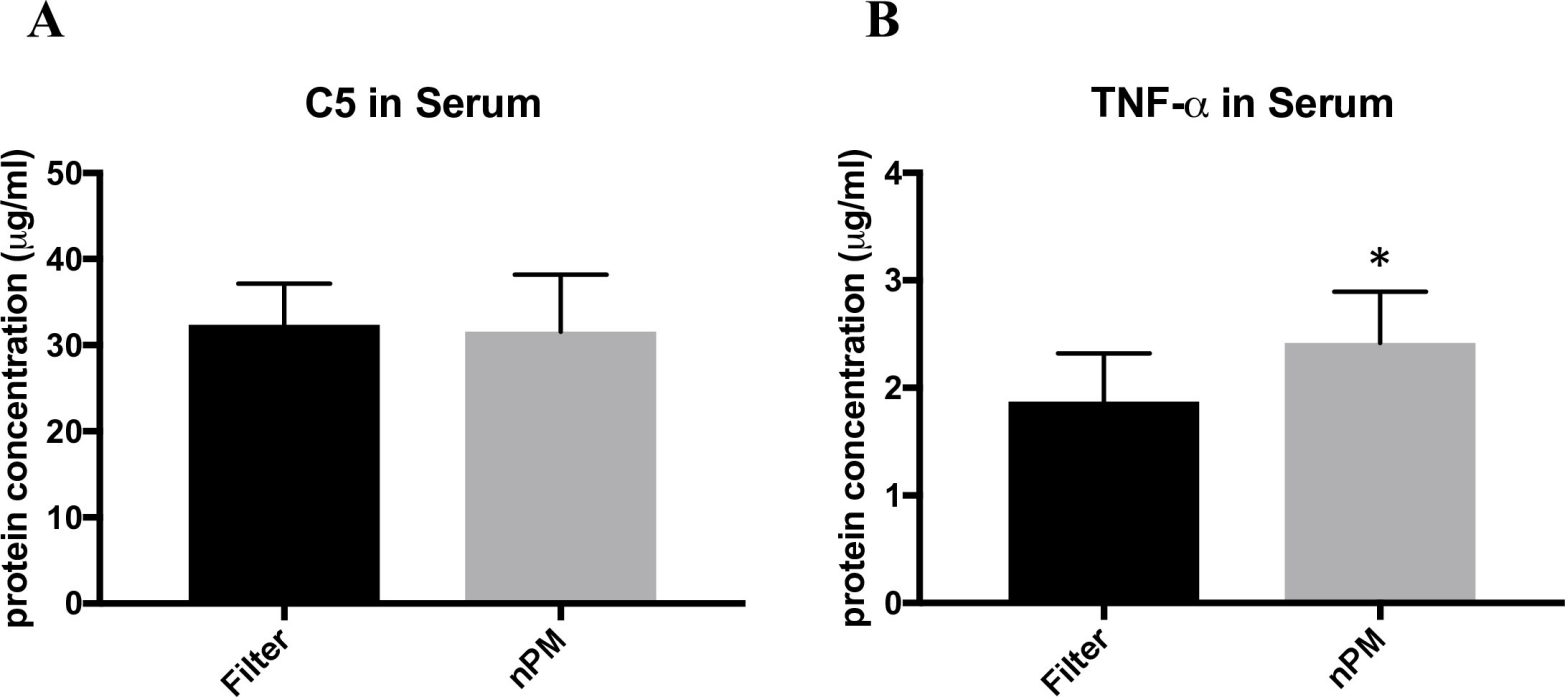
ELISA of serum C5 and TNF-alpha in animals exposed to filtered air or nanoparticulate matter (nPM). Protein concentrations expressed as log values. (A) Serum C5 levels did not differ between the nPM and filtered air groups (p = 0.785). (B) TNF-alpha levels were significantly elevated in the nPM group compared to the filtered air group (p = 0.0419). * signifies p< 0.05. Error bars represent standard deviation.

Serum ELISA C5 values (ηg/mg total protein) did not significantly differ between filtered air (32.4 ± 4.8, n = 8) and nPM mice (31.6 ± 6.6, p = 0.79, n = 8). See Fig 4A. However, Serum ELISA TNF-alpha values (ηg/mg total protein) were elevated 28% in nPM mice (2.4 ± 0.50, n = 8) compared to filtered air mice (1.9 ± 0.5, p = 0.04, n = 7) (Fig 4B).

## Discussion

Clinical and epidemiological investigations have demonstrated strong associations between air pollution particulate matter exposure and neurocognitive injury[26–29]. Both laboratory and translational studies suggest white matter pathology as an anatomic correlate for particulate matter induced neuropsychological decline and advocate an inflammatory mechanism of action. Data from the present study demonstrates increased immunostaining of complement C5 protein, C5a receptor 1 (CD 88), and reactive microglia in the brain white matter (corpus callosum) of mice exposed to nPM. Previously published results suggest that astrocytes, neurons, and microglia express CD88, while only astrocytes and neurons express C5 [30, 31]. Our co-staining analysis suggests that neurons express C5 following air pollution exposure [30, 31]. C5 and GFAP positive cells did not precisely co-localize, so C5 may be produced from astrocytes or the surrounding neurons. Our IBA-1 and C5 immunostaining appeared to co-localize, suggesting either microglial or nearby neuronal expression of C5. C5 may be binding to CD88, resulting in this co-localization or microglia may produce increased levels of C5 following particulate matter exposure.

The C5 complement immunostaining likely results from a local response within the brain rather than upregulation of complement in the blood and entrance through the blood brain barrier. While both C5 and C5α staining densities were greater in the white matter of mice exposed to nPM, serum C5 levels did not differ between the groups (nPM vs. filtered air). By contrast, TNF-alpha levels were greater in the nPM-exposed mice, supporting a generalized peripheral inflammatory response that is well recognized in the setting of nPM exposure[32–34].

Prior studies demonstrate that diesel exhaust particles activate the complement cascade, particularly the anaphylatoxins, C3a and C5a[35]. Complement modulation (C3 deficiency) has, in turn, been leveraged to decrease exposure-generated inflammation in a murine model of particulate matter-induced lung/ airway hyperresponsiveness[36]. These findings may be generally applicable to other organ systems, including the brain [37]. Our results demonstrate the importance of the complement C5 component in brain white matter inflammation following particulate matter (PM2.5) exposure and specifically focus on the role of microglia. C5a, a chemotaxin for leukocytes and inflammation, induces calcium mobilization and enhances microglial activation[38]. In vitro studies demonstrate transient calcium elevations in microglia on the tissue surface of corpus callosum slices exposed to C5a complement fragments. The investigators observed calcium elevations in microglial cell cultures that were supplemented with complement C5a and C3a[16]. In the resting state, microglia are characterized by small cell bodies, roughly 2–5 μm in diameter [39, 40] with long, branching processes [24, 41]. When microglia become activated, their cell bodies enlarge and their dendritic processes shorten and thicken [24, 42–44]. In the present study, the increased cell body to dendritic process ratio following air pollution exposure demonstrates a change in microglial morphology, characteristic of activated microglia[24].The interplay between complement C5 and reactive microglia represents a logical effector of neuroinflammation/ neurotoxicity in our model system, as microglial activation and production of potentially injurious byproducts have been previously demonstrated in the setting of particulate matter exposure. In vitro nanosize titanium dioxide exposure stimulates microglial production of reactive oxygen species[45]. These findings are consistent with human data. Autopsy studies of individuals exposed to high levels of air pollution in Mexico City have shown increased CD14 expression, a marker of both resident microglial cells and infiltrating monocytes[46].

Previous data supports a specific role for complement receptor-mediated microglial activation in the setting of diesel exhaust exposure[47, 48]. MAC-1 receptor (complement receptor 3) is expressed exclusively on myeloid lineages, such as microglia. The MAC-1 receptor facilitates reactive microgliosis in response to multiple neurotoxins, including MPTP[49], LPS[50], and alpha-synuclein [51]. Levesque et al have demonstrated that MAC1 is an essential receptor in the process of H_2_O_2_ production by activated microglia following DEP exposure[47]. Complement component C5a receptor 1 (CD 88) is known to play a critical role in the calcium signaling required for phagocytosis in microglia[52]. It is constitutively expressed on multiple glial cell lines[14]; however, CNS CD88 expression is influenced by inflammation[53–58], of which particulate matter is a potent source. Mixed neuron-glia cultures exposed to nanosized diesel exhaust particles produce dopaminergic neurotoxicity only in the presence of microglia[59]. Similarly, our data demonstrates a significant increase in corpus callosum CD88 staining and reactive microglia in mice exposed to nPM.

Block et al. suggest that CNS pathology secondary to nPM exposure results from a combination of soluble compounds reaching the brain and peripheral mechanisms involving circulating cytokines from a systemic inflammatory response with the ultimate activation of microglia [7]. In our model system, brain complement C5 deposition could be triggered either by direct nPM toxicity (through the olfactory bulb or blood brain barrier) or a systemic inflammatory response that permeates the blood brain barrier and upregulates complement production via endogenous neurons or glia. Enhanced complement deposition/ upregulation, in turn, may affect microglial migration or activation via CD88 receptors on primarily microglia.

The neuroinflammatory response evident in the corpus callosum in our model appears to be microglia-specific as the nPM cohort showed increases in both cell count and density, as well as a change in morphology, consistent with activated microglia [24]. No significant differences in cell count and cell density of reactive astrocytes were observed between the two groups (nPM and filtered air). The relative lack of astrocytic reactivity in our experiments is supported by a study demonstrating that GFAP levels are unaltered in the brains of rats exposed to combustion products derived from burning of 100% soy bean oil [60]. By contrast, increased GFAP reactivity has been demonstrated following air pollution exposure in the gray matter of the developing brain [61, 62]. Our model focused on the effect of particulate matter in the adult brain, specifically the corpus callosum. The developing brain is more susceptible to injury[63], but also has greater plasticity [64], which may allow for faster recovery. In addition, white matter contains myelin and is suggested to be more susceptible than gray matter to certain types of injury, such as ischemia [65–67]. Calderón-Garcidueñas and colleagues also found that canines exposed to highly polluted air in Mexico City exhibited increased astrocyte reactivity, particularly in white matter, when compared to canines from a less polluted environment[68]. The air pollution in the Mexico City study had relatively higher ozone, PM2.5 and aldehydes than the Los Angeles air sample in our exposure paradigm. Additional environmental factors may have also impacted results, as subjects in the studies by Calderon-Garciduenas et al [34, 46, 68] were not separated directly by air pollution exposure, but rather by geographical location.

White matter damage has been linked to complement upregulation and microglial activation in other experimental systems of neuroinflammation. In the autoimmune encephalitis (EAE) model of multiple sclerosis (MS), microglial cells were found to participate in early stages of myelin destruction[69]. C5 deficient mice demonstrated greater axonal preservation and myelin formation following EAE[70]. In an LPS model of oligodendrocyte death, Li et al. mechanistically demonstrated a role for microglial-derived peroxynitrite, a deleterious byproduct of NADPH oxidase, suggesting that activated microglia could play a critical role in white matter disorders[71]. An investigation of post-mortem brains of healthy children residing in highly polluted environments demonstrated significant staining with CD 68 (a microglial marker) in white matter regions[46]. In an AD transgenic mouse model, Hong et al found that microglia engulf synaptic material in a complement dependent process when exposed to soluble Aβ oligomers[72]. Taken together, these findings suggest that critical interactions between complement components and activated macrophages/ microglia may play an important role in the white matter response to a host of neuroinflammatory states.

The fact that our cohort consisted of exclusively male mice is a potential limitation of this study. Previous studies have suggested that estrogen may have a protective effect against white matter injury [61, 73]. This effect, particularly in the setting of inflammatory damage, may be explained by microglial expression of estrogen receptors. It is hypothesized that estrogen binding serves to prevent microglial activation and resulting injury[74]. Future studies investigating air pollution exposure in female mice can be leveraged to assess the sex differences and estrogen’s role in white matter damage. The potential effects of the anesthetic regimen at the time of euthanasia must also be considered when interpreting the cellular data. Given the findings of previous studies[75–77], it is unlikely that ketamine-xylazine anesthesia significantly altered the levels of inflammation.

The size and chemical composition of the particulate matter used in this experiment are comparable to that present in an urban area. nPM exposure levels of approximately 330 μg/m^3^ are roughly twice the levels present on busy Los Angeles freeways with a high volume of diesel trucks [21, 78, 79]. This concentration is similar to those used in previous studies [42, 60, 80].

In conclusion, this study demonstrates increased complement C5 immunostaining and microglial activation secondary to nPM exposure. The complement upregulation appears to be localized to the brain, as serum C5 levels did not differ between nPM and filtered air-exposed mice. These findings indicate that interaction between the complement anaphylatoxins (particularly C5a) and activated microglia may play an important role in white matter injury following nPM exposure.

## Supporting information

S1 File IBA-1 Cell count and density dataset. (XLSX)

S2 File IBA-1 Cell body and dendritic process size datasheet. (XLSX)

S3 File GFAP cell count and density dataset. (XLSX)

S4 File C5 and C5α density dataset. (XLSX)

S5 File CD88 density dataset. (XLSX)

S6 File Serum C5 and serum TNF-alpha dataset. (XLSX)
